# Alcohol and Exercise Affect Declining Kidney Function in Healthy Males Regardless of Obesity: A Prospective Cohort Study

**DOI:** 10.1371/journal.pone.0134937

**Published:** 2015-08-03

**Authors:** Eiichiro Kanda, Toshitaka Muneyuki, Kaname Suwa, Kei Nakajima

**Affiliations:** 1 Department of Nephrology, Tokyo Kyosai Hospital, Meguro, Tokyo, Japan; 2 Center for life science and bioethics, Tokyo Medical and Dental University, Bunkyo, Tokyo, Japan; 3 Saitama Citizens Medical Center, Saitama, Saitama, Japan; 4 Department of Rehabilitation, Funabashi City Rehabilitation Hospital, Funabashi, Chiba, Japan; 5 Saitama Health Promotion Corporation, Hikigun, Saitama, Japan; 6 Division of Clinical Nutrition, Department of Medical Dietetics, Faculty of Pharmaceutical Sciences, Josai University, Sakado, Saitama, Japan; 7 Department of Metabolism, Kuki General Hospital, Kuki, Saitama, Japan; Aichi Cancer Center Research Institute, JAPAN

## Abstract

**Background:**

Although lifestyle is associated with metabolic syndrome and cardiovascular diseases, there has been no sufficient evidence of lifestyles on incident chronic kidney disease (CKD). The purpose of this prospective cohort study is to investigate the effects of lifestyles on kidney function in healthy people.

**Methods:**

A total of 7473 healthy people were enrolled in this Saitama Cardiometabolic Disease and Organ Impairment Study, Japan. Data on alcohol consumption, exercise frequency, and sleep duration were collected. The outcome event was incident CKD or decrease in estimated glomerular filtration rate (eGFR) by >25% in 3 years.

**Results:**

Subjects were classified into four groups according to body mass index and gender. Mean ± standard deviation of age was 38.8±10.5 years; eGFR, 78.1±15.2 ml/min/1.73m^2^. In the male groups, multivariate logistic regression models showed that the outcome events were associated with a small amount of alcohol consumed (20 to 140g of alcohol/week) (ref. more than 140g of alcohol/week); non-obese male, adjusted odds ratio 1.366 (95% confidence interval, 1.086, 1.718); obese male (body mass index ≥25), 1.634 (1.160, 2.302); and with frequent exercise (twice a week or more) (ref. no exercise); non-obese male, 1.417 (1.144, 1.754); obese male, 1.842 (1.317, 2.577). Sleep duration was not associated with the outcome events.

**Conclusion:**

These findings suggest that, regardless of obesity, a small amount of alcohol consumed and high exercise frequency were associated with the increased risk of loss of kidney function in the male groups.

## Introduction

It has been reported that in chronic kidney disease (CKD) patients some lifestyles have combined effects on the life prognosis of these patients [1]. Moreover, lifestyles are related to metabolic syndrome, which is a factor related not only to diabetes mellitus (DM) and cardiovascular diseases (CVDs) but also to incident CKD [2]. However, there has been no sufficient evidence of the effects of lifestyles on incident CKD.

The relationship between an amount of alcohol consumed and incident CKD has been investigated in longitudinal observational studies. In a community-based followed-up study carried out in Japan, the risk of incident CKD decreased in the group with alcohol consumption of less than 20 g/day but did not significantly decrease in the group with alcohol consumption of 20 g/day or more compared with that in the non-alcohol-consuming group [3]. According to the Australian population-representative study (AusDiab study), the risk of incident CKD are reduced in the group with a large amount of alcohol consumed [4].

In a systematic review, improvements in aerobic capability, muscular function, cardiovascular function, walking capacity, and health-related quality of life were reported as the effects of exercise in CKD patients [5]. The protective effects of exercise on glomerular filtration rate (GFR) were not demonstrated in interventional studies on CKD patients owing to the small sample size [6, 7]. In a cross-sectional study conducted by the National Health and Nutrition Examination Survey (NHANES), the hours of sleep are shorter in the patients with CKD stages 1 and 2 than in those with CKD stages 3 and 4 [8]. As far as we investigated, there have been no longitudinal studies clarifying the relationship between hours of sleep and incident CKD.

Obesity and metabolic syndrome have been reported as risk factors for CKD [9]. The gender difference in the relationship between body mass index (BMI) and CKD has been reported [10]. Obese females have a higher risk of CKD than non-obese females, and there is no difference in the risk of CKD between obese and non-obese males. To prevent metabolic syndrome and DM, exercise is often recommended for obese people to lose weight. On the other hand, non-obese people do not need to lose weight. When lifestyle guidance is provided to healthy people in order to prevent CKD, the content of the guidance is determined by their gender and obesity. However, there has been no report on the effects of lifestyle on the kidney function of healthy people, with both their gender and obesity simultaneously taken into consideration.

The Saitama Cardiometabolic Disease and Organ Impairment Study (SCDOIS) in Japan is a community-based longitudinal observational study [11, 12]. In this study, community-based data were collected over 9 years from medical checkups of asymptomatic people. The purpose of this study was to clarify the relationship of lifestyles (an amount of alcohol consumed, exercise frequency, and sleep duration) with decrease in estimated GFR (eGFR) and incident CKD considering the differences in gender and obesity among subjects on the basis of the SCDOIS data.

## Materials and Methods

### Data Source

SCDOIS was a multidisciplinary observational epidemiological research study in Saitama Prefecture, Japan [11, 12]. In brief, this study started in 2011. The protocol was in accordance with the Declaration of Helsinki and was approved by the Ethics Committees of Josai University and Saitama Health Promotion Corporation. Written informed consent was obtained at the time of the checkup from all the subjects. However, because the informed consent from the subjects was not obtained concerning the availability of their data to the public, there were restrictions on sharing of data.

**Fig 1:**
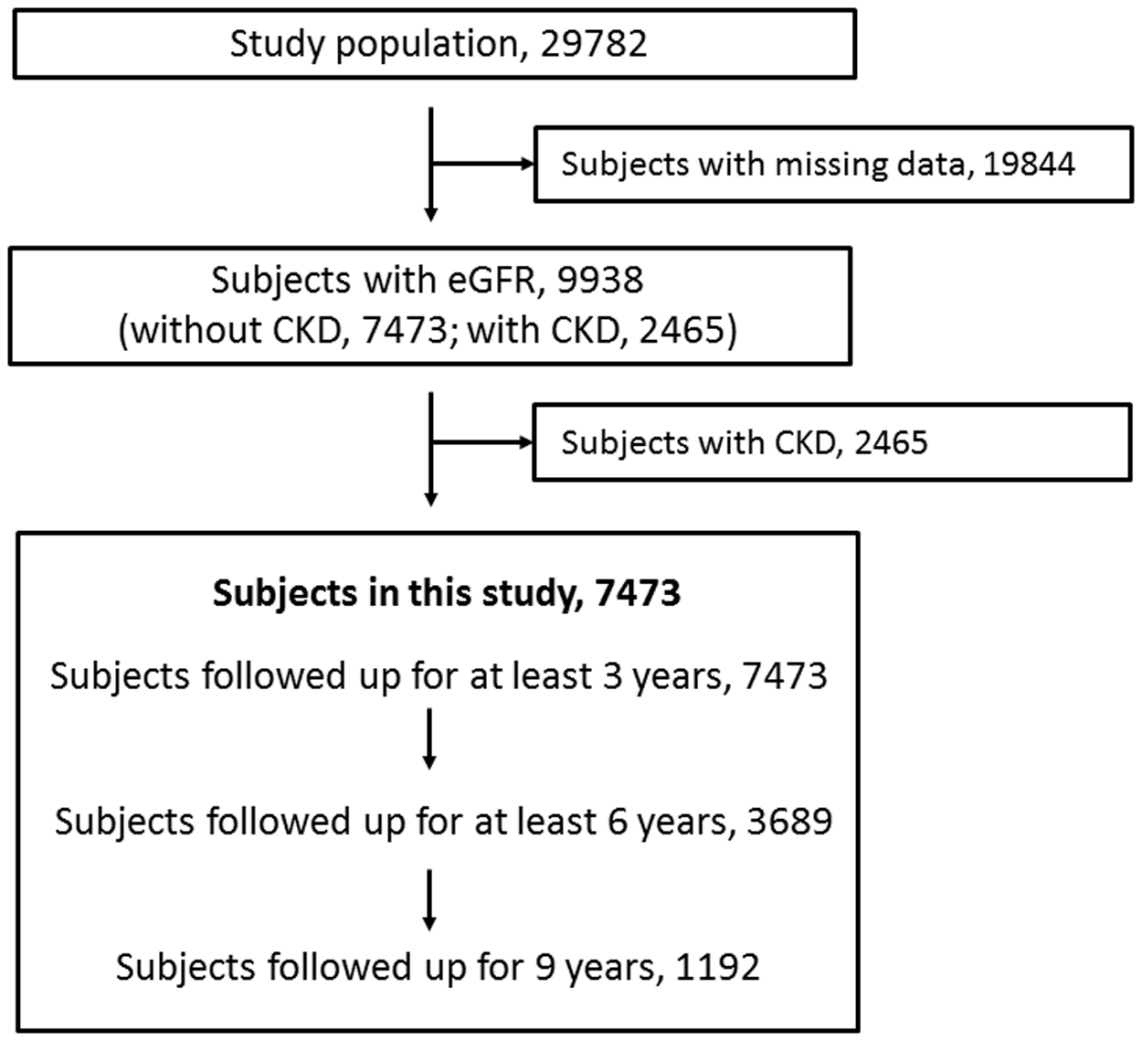
Flow diagram of subject assignment. Subjects were followed up for 3 to 9 years. The analysis was conducted on 7473 subjects followed up for at least 3 years, 3689 for at least 6 years, and 1192 for 9 years. That is, the 3784 subjects had two-times-measured data (baseline and 3-year followed-up data); 2497 subjects, three-times-measured data (baseline, 3- and 6-year followed-up data); and 1192 subjects, four-times-measured data (baseline, 3-, 6-, and 9-year followed-up data).

This study involved the analysis of data collected every three years from the records of medical checkups of asymptomatic people living or working in Saitama from 1999 to 2008. The study population consisted of 29782 subjects, who were followed up for at least 3 years (Fig 1). Subjects with missing data such as age, gender, and serum creatinine level were excluded from this study. The number of participants from whom eGFR data were available was 9938. Among them, 2465 subjects with CKD were excluded. The following numbers of subjects were included in this study: 7473 followed up for at least 3 years; 3689 for at least 6 years; and 1192 for 9 years. That is, of 7473 subjects, 3784 subjects had two-times-measured data (baseline and 3-year followed-up data); 2497 subjects, three-times-measured data (baseline, 3- and 6-year followed-up data); and 1192 subjects, four-times-measured data (baseline, 3-, 6-, and 9-year followed-up data). The data of these 7473 subjects were treated as baseline data.

Baseline data including age, gender, BMI, serum creatinine levels, urinary protein excretion, comorbid conditions of diabetes mellitus, hypertension, and dyslipidemia, histories of CVDs, smoking, alcohol consumption, exercise frequency, and sleep duration were collected from all the subjects. Data were collected every three years. eGFR was calculated using the following equation of the Japanese Society of Nephrology [13]: eGFR (ml/min/1.73m^2^) = 194×serum Cr^-1.094^×age^-0.287^ (for female) ×0.739, where Cr = serum creatinine level (mg/dl). Annual GFR decline speed was calculated using the following formula: eGFR decline speed = (eGFR_after_—eGFR_before_) / 3 (ml/min/1.73m^2^/year). The minus and plus values of eGFR decline speed indicated that eGFR was decreasing and increasing, respectively. To evaluate the effects of lifestyles on the loss of kidney function of subjects with both their gender and obesity simultaneously taken into consideration, subjects were categorized by gender and BMI. BMI was used to categorize the subjects as follows: non-obese, less than 25; obese, 25 or more. An amount of alcohol consumed in a week (g of alcohol/week) was measure based on glasses of Sake and calculated as follows: an amount of alcohol consumed in a day (g of alcohol/day) × days in a week, where 1 glass (go) of Sake = 20g of alcohol. The categories of alcohol consumption were as follows: 0 (no alcohol consumed), alcohol consumption = 0; 1 (a small amount of alcohol consumed) = 20 to 140g of alcohol/week; 2 (a large amount of alcohol consumed) = more than 140g of alcohol/week. Exercise was defined as a more than 30-minute exercise with sweating. The categories of exercise frequency were as follows: 0, twice a month or less; 1, once a week; 2, twice a week or more. The categories of sleep duration were as follows: 0, 6 hours or less; 1, 7 hours; 2, 8 hours or more.

### Statistical analyses

The subjects were classified into four groups according to BMI and gender: non-obese male, obese male, non-obese female, and obese female. Statistical analyses were carried out separately for the obese-gender groups. Data are presented as mean ± standard deviation. Intergroup comparisons were performed using the chi-square test, one-way analysis of variance, and Kruskal-Wallis one-way analysis of variance as appropriate. For variables not normally distributed, natural logarithm values were considered: natural logarithm values of an amount of alcohol consumed [ln(alcohol)]. For the calculation of ln(alcohol) for non-alcohol drinkers, their amount of alcohol consumed was treated as 1 g of alcohol/week. An outcome event was defined as incident CKD or decrease in eGFR by more than 25% within three year. The outcome events were evaluated using the 3-year data of 7473 subjects. Multivariate logistic regression models adjusted for age, gender, BMI, eGFR, urinary protein excretion, comorbid conditions of diabetes mellitus, hypertension and dyslipidemia, histories of CVDs, and smoking were used to examine the association between categories of lifestyles (categories of alcohol consumption, exercise frequency, and sleep duration) and the outcome events. Results were presented as adjusted odds ratio (aOR) with 95% confidence interval (CI). To examine the relationships of eGFR decline speed with an amount of alcohol consumed, exercise frequency, and sleep duration, multivariate linear mixed models were used for the repeatedly measured data. The models were adjusted for age, gender, BMI, eGFR, urinary protein excretion, comorbid conditions of diabetes mellitus, hypertension and dyslipidemia, histories of CVDs, and smoking. Age, BMI, and eGFR were used as time-dependent variables in the models. Statistical analyses were performed using SAS version 9.2 (SAS Institute, Cary, NC). Values of *p* < 0.05 were considered statistically significant.

## Results

### Baseline characteristics

Subjects’ demographics including biochemical data are shown in Table 1. The numbers of people in the obese groups with urinary protein excretion; comorbid conditions of diabetes mellitus, hypertension and dyslipidemia, and histories of CVDs were larger than those in the non-obese groups. More people with diabetes mellitus and who smoke were observed in the male groups than in the female groups. The obese female group was older and showed lower eGFR than the other groups.

**Table 1:** Baseline characteristics.

	All	Non-obese male group	Non-obese female group	Obese male group	Obese female group	*p*
N (%)	7473	3910 (52.3)	1625 (21.7)	1662 (22.3)	276 (3.7)	
Age	38.8±10.5	38.8±10.5	37.7±10.9	39.3±10.0	41.3±10.6	0.0001
BMI	23.2±3.4	22.0±10.5	20.9±2.0	27.6±2.6	27.9±2.6	0.0001
eGFR (ml/min/1.73m^2^)	78.1±15.2	78.7±15.3	78.8±16.3	76.4±13.6	74.8±13.9	0.0001
Urinary protein excretion (%)	296 (4.0)	124 (3.2)	47 (2.9)	114 (6.9)	11 (4.0)	0.0001
CKD stage						0.0001
Stage 1	1529 (20.5)	851 (21.8)	379 (23.3)	261 (15.7)	38 (13.8)	
Stage 2	5944 (79.5)	3059 (78.2)	1246 (76.7)	1401 (84.3)	238 (86.2)	
Diabetes mellitus	188 (2.5)	107 (2.7)	9 (0.6)	66 (4.0)	6 (2.2)	0.0001
Hypertension	316 (4.2)	133 (3.4)	36 (2.2)	118 (7.1)	29 (10.5)	0.0001
Dyslipidemia	325 (4.3)	173 (4.4)	47 (2.9)	92 (5.5)	13 (4.7)	0.0028
CVD	137 (1.8)	64 (1.6)	20 (1.2)	39 (2.3)	14 (5.1)	0.0001
Smoking	3025 (40.5)	1963 (50.2)	184 (11.3)	850 (51.1)	28 (10.1)	0.0001
Amount of alcohol consumed (g of alcohol/week)	71.4±109, 20 (0, 140)	92.9±117.7, 40 (0, 140)	20.1±51.3, 20 (0, 80)	91.2±119.2, 40 (0, 140)	11.9±34.1, 0 (0, 0)	0.0001
Category of alcohol consumption (%)						0.0001
0	3575 (47.9)	1489 (38.1)	1230 (75.7)	635 (38.2)	221 (80.1)	
1	2686 (35.9)	1605 (41.0)	363 (22.3)	666 (40.1)	52 (18.8)	
2	1212 (16.2)	816 (20.9)	32 (2.0)	361 (21.7)	3 (1.1)	
Category of exercise frequency (%)						0.0001
0	4676 (62.6)	2264 (57.9)	1221 (75.1)	992 (59.7)	199 (72.1)	
1	1382 (18.5)	777 (19.9)	232 (14.3)	330 (19.9)	43 (15.6)	
2	1415 (18.9)	869 (22.2)	172 (10.6)	340 (20.4)	34 (12.3)	
Sleep duration (hours)	6.7±0.9	6.7±0.9	6.5±0.9	6.6±0.9	6.6±0.9	0.0001
Category of sleep duration (%)						0.0001
0	3442 (46.1)	1637 (41.9)	888 (54.6)	769 (46.3)	148 (53.6)	
1	2864 (38.3)	1582 (40.4)	546 (33.6)	648 (39.0)	88 (31.9)	
2	1167 (15.6)	691 (17.7)	191 (11.8)	245 (14.7)	40 (14.5)	
Incident CKD (%)	1610 (21.5)	665 (17.0)	486 (29.9)	361 (21.7)	98 (35.5)	0.0001
Decrease in eGFR (>25%) (%)	2208 (29.56)	1093 (28.0)	609 (37.5)	415 (25.0)	91 (33.0)	0.0001
Outcome event (%)	2809 (37.6)	1366 (34.9)	765 (47.1)	555 (33.4)	123 (44.6)	0.0001

Values are presented as mean±SD. The amount of alcohol consumed are presented mean±SD and median (first and third quartiles) values.

Abbreviations: BMI, body mass index; eGFR, estimated glomerular filtration rate; CKD, chronic kidney disease; CVD, cardiovascular disease; outcome event, incident CKD or decrease in eGFR (>25%).

The amount of alcohol consumed and exercise frequency were higher in the male groups than in the female groups. The sleep duration was longer in non-obese males than in other groups. The incidences of both incident CKD and decrease in eGFR were higher in the non-obese female group than in the non-obese male group, and higher in the obese female group than in the obese male group. The incidences of outcome events were higher in the female groups than male groups. There were slight differences in the outcome events between the non-obese male group and the obese male group, and between the non-obese female group and the obese female group.

### Lifestyles and loss of kidney function

**Fig 2:**
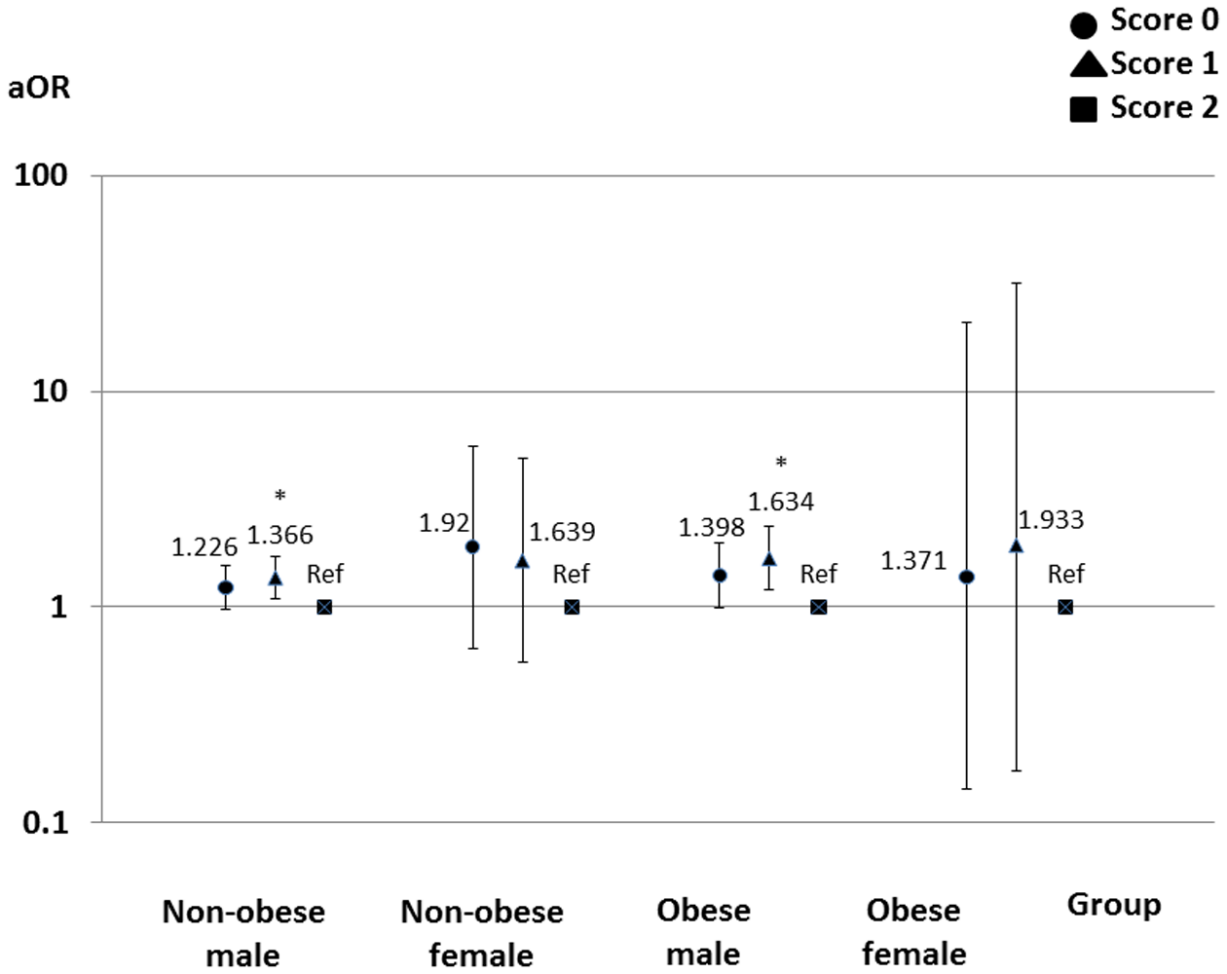
Effects of alcohol consumption on loss of kidney function. In males, the small amount of alcohol consumed was more associated with loss of kidney function than the large amount of alcohol consumed. Score 0 = no alcohol consumed; score 1 (a small amount of alcohol consumed) = 20 to 140g of alcohol/week; score 2 (a large amount of alcohol consumed) = more than 140g of alcohol/week. Adjusted odds ratios are shown with 95% confidence intervals. The logistic regression models are adjusted for the baseline characteristics. *, *p*<0.05. Abbreviations: aOR, adjusted odds ratio; Ref, reference.

**Fig 3:**
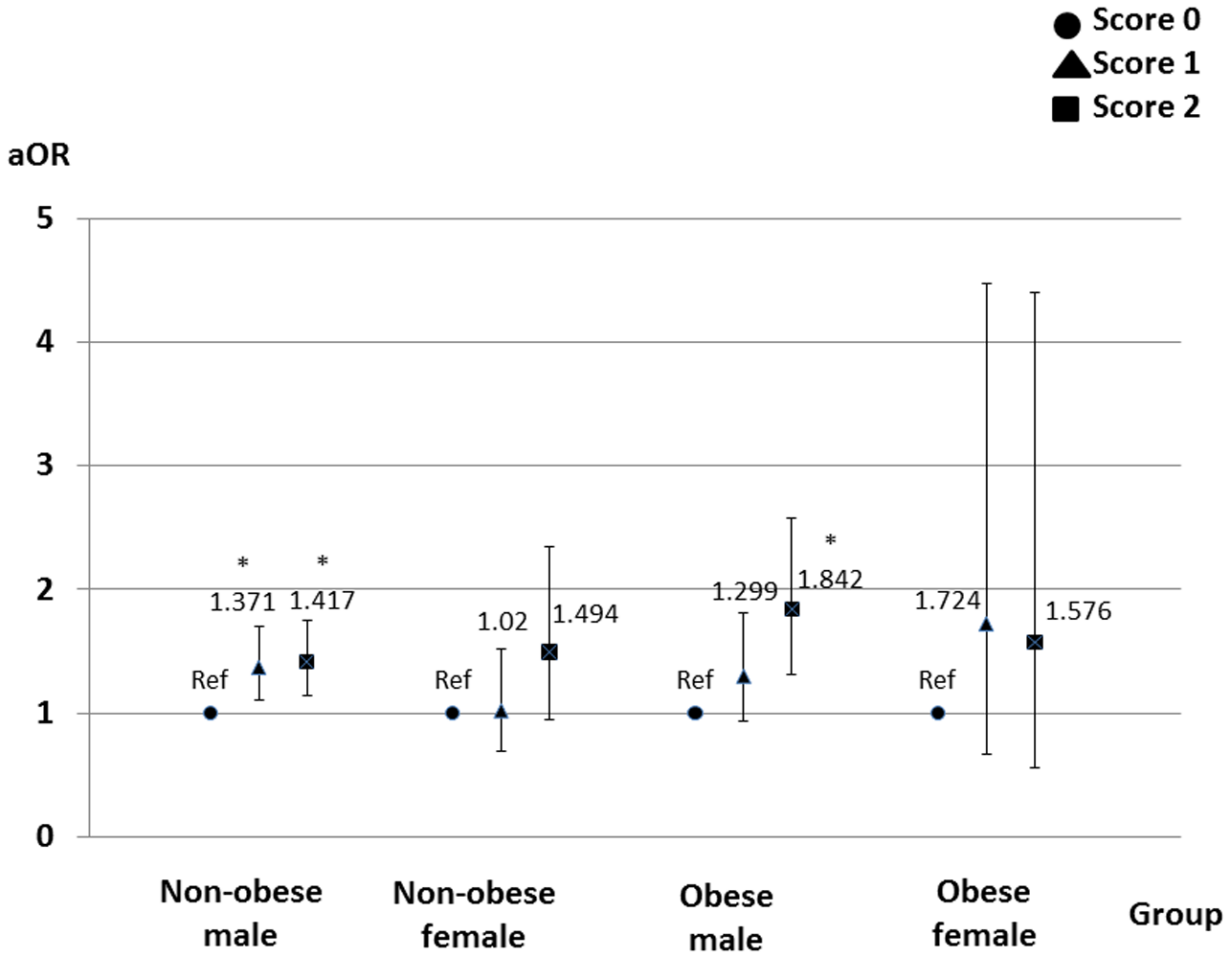
Effects of exercise frequency on loss of kidney function. In males, frequent exercise was more associated with loss of kidney function than no exercise. Score 0 = twice a month or less; score 1 = once a week; score 2 = twice a week or more. Adjusted odds ratios are shown with 95% confidence intervals. The logistic regression models are adjusted for the baseline characteristics. *, *p*<0.05. Abbreviations: aOR, adjusted odds ratio; Ref, reference.

**Fig 4:**
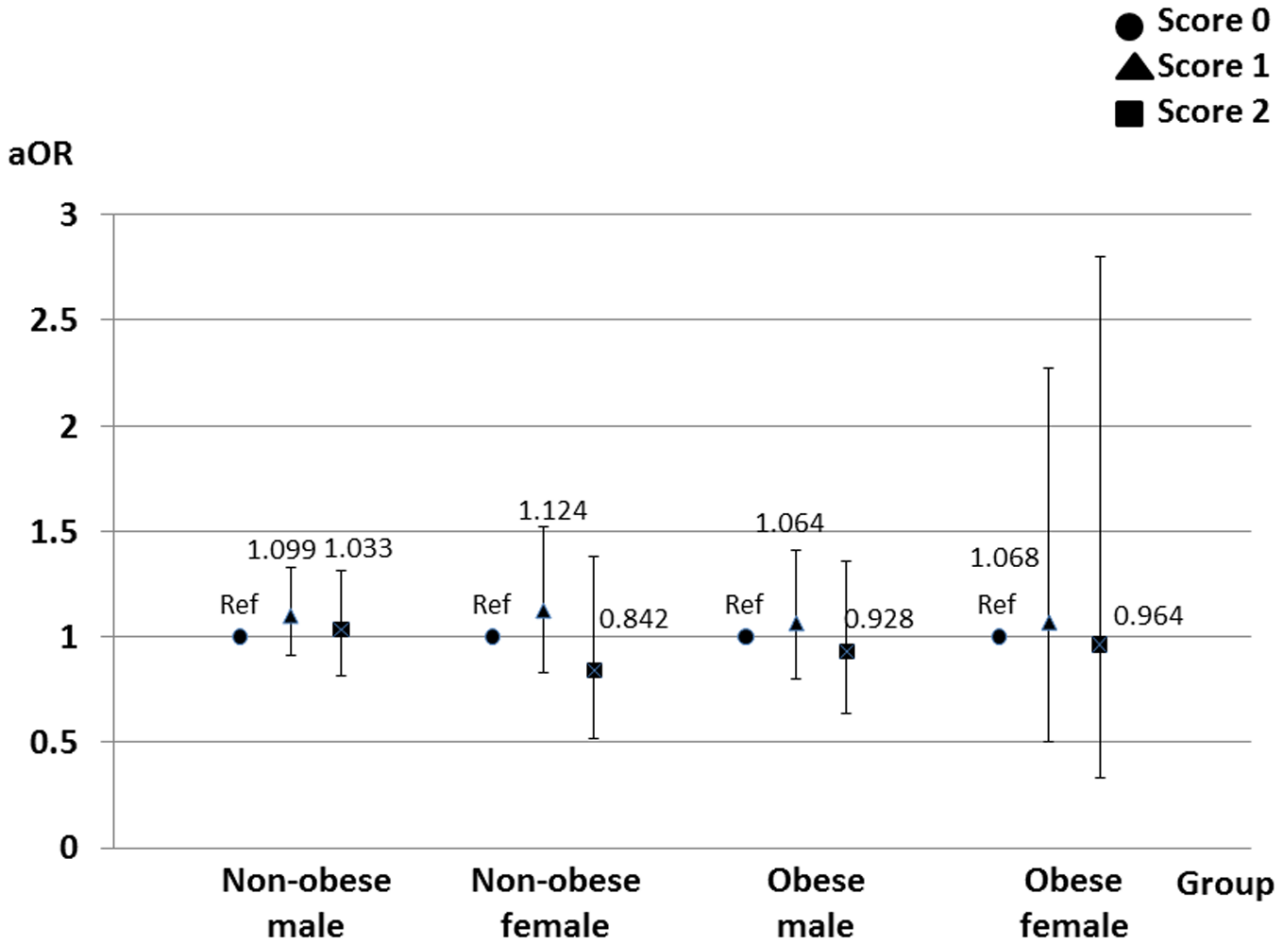
Effects of sleep duration on loss of kidney function. Sleep duration was not associated with loss of kidney function. Score 0 = 6 hours or less; score 1 = 7 hours; score 2 = 8 hours or more. Adjusted odds ratios are shown with 95% confidence intervals. The logistic regression models are adjusted for the baseline characteristics. *, *p*<0.05. Abbreviations: aOR, adjusted odds ratio; Ref, reference.

Multivariate logistic regression models showed that the outcome events were more associated with the small amount of alcohol consumed than the large amount of alcohol consumed, and with frequent exercise than no exercise in the male groups (Figs 2 and 3). The sleep duration was not associated with the outcome events (Fig 4).

### Lifestyles and eGFR decline speed

Multivariate linear mixed models showed that eGFR decline speed positively correlated with ln(alcohol) in the male groups (Table 2). eGFR decline speed negatively correlated with exercise frequency in the non-obese male and non-obese female groups, and marginally in the obese male and obese female groups. That is, decrease in eGFR was associated with decrease in ln(alcohol) in the male groups, and with increase in exercise frequency. Sleep duration did not correlate with eGFR decline speed.

**Table 2:** Association between lifestyles and eGFR decline speed.

	Non-obese male group	Non-obese female group	Obese male group	Obese female group
Ln(alcohol)	0.0925 (0.0377) *p* = 0.014	0.0897 (0.0795) *p* = 0.260	0.204 (0.061) *p* = 0.0009	-0.203 (0.250) *p* = 0.42
Exercise frequency (1 increase in the score)	-0.491 (0.104) *p* = 0.0001	-0.847 (0.214) *p* = 0.0001	-0.326 (0.170) *p* = 0.056	-0.949 (0.519) *p* = 0.072
Sleep duration (hours)	0.0821 (0.104) *p* = 0.43	0.132 (0.170) *p* = 0.44	0.158 (0.167) *p* = 0.34	0.182 (0.438) *p* = 0.68

Decrease in eGFR was associated with increase in ln(alcohol) in the male groups, and with increase in exercise frequency.

Values are given as parameter estimates (standard error) and *p* values. The minus and plus values of parameter estimates indicate that eGFRs were decreasing and increasing, respectively. Multivariate linear mixed models were adjusted for baseline characteristics. Age, BMI, and eGFR were treated as time-dependent variables.

Abbreviations: ln(alcohol), natural logarithm values of an amount of alcohol consumed; BMI, body mass index; eGFR, estimated glomerular filtration rate.

## Discussion

In this study, the healthy people were classified into groups according to their gender and obesity in order to clarify the relationship between the loss of kidney function and lifestyles. In the male groups, regardless of obesity, the loss of kidney function less likely occurred in the subjects with the larger amount of alcohol consumed and more likely occurred in the groups with higher exercise frequency. In the female groups, the amount of alcohol consumed and exercise frequency did not correlate with the loss of kidney function. No correlation between sleep duration and the loss of kidney function was observed in any of the groups.

The results of this study showed that the kidney function less likely declined with the large amount of alcohol consumed in the male groups and that the amount of alcohol consumed was not associated with the loss of kidney function in the female groups regardless of obesity. These findings were in agreement with the results of previous studies [3, 4, 14–16]. Regarding the mechanism underlying the protective effect of alcohol consumption on kidney function, it was reported that alcohol consumption prevents the hyalinization of renal arterioles [17]. Alcohol consumption increases the high-density lipoprotein cholesterol (HDL) level [18]. In the Atherosclerosis Risk in Communities study, it was shown that a low HDL cholesterol level increases the probability of developing kidney dysfunction [19]. A randomized controlled trial showed that both wine and gin have anti-inflammatory effects by reducing plasma fibrinogen and IL-1alpha levels [20]. Polyphenol in flavonol-rich red wine has a protective effect on the kidney in rats [21, 22]. A randomized cross-over trial showed that red wine intake has antioxidant effects due to polyphenol [23]. Moreover, it has been reported that a large amount of alcohol consumed (231 g or more of alcohol/week in males) is associated with less coronary atherosclerosis and a lower risk for cardiac mortality than a small amount of alcohol consumed (less than 231 g of alcohol/week in males) or no consumption [24]. These mechanisms may more strongly affect kidney function in males than in females by the prevention of atherosclerosis.

It has been reported that estrogen affects the glomerular and vascular remodeling and induces cardiorenal protection [25]. Estrogen prevents CKD progression by lowering the cardiovascular stress response to adrenergic stimuli [26]. Moreover, it has been reported that testosterone induces apoptosis in proximal tubule cells [27]. Alcohol consumption increases the estrogen level in females [28, 29] and decreases testosterone levels [30]. These findings suggest that alcohol consumption may modify the effect of sex hormones on the loss of kidney function.

The relationship between an amount of alcohol consumed and kidney function varied from study to study. In a cohort study of the elderly (Cardiovascular Health Study), an amount of alcohol consumed did not correlate with the decrease in eGFR [31]. In a community-based study in Japan, the reduction of risk of incident CKD due to alcohol consumption was not observed in males (mean age, 61.8 years) at alcohol consumption of 20 g/day or more [3]. In the AusDiab study, the risk of incident CKD decreased in the males (younger than 65 years) who were moderate and heavy drinkers [4]. In a study of healthy males (mean age, 52.4 years), the risk of decrease in GFR was reduced in the group drinking alcohol five or more times a week [15]. The mean age of the males in our study, approximately 39 years, was younger than that in other studies. Alcohol metabolism tends to decrease in the elderly [32], which suggests that the effect of alcohol consumption in terms of reduction of the risk of loss of kidney function changes with age.

The results of this study indicate that exercise frequency was associated with the loss of kidney function in the male groups. There has been a report regarding the decrease in renal cortical blood flow due to exercise [33], which indicated that exercise may lead to loss of kidney function depending on the type and an amount of exercise. Moreover, weight loss is not always beneficial for kidney function. A cohort study showed a u-shaped association between weight change and incident CKD among healthy males with normal weight [34]. Another cohort study showed that the percent change in BMI (< 1%) is associated with incident CKD in males [35]. These studies suggest that weight loss may not always be beneficial to kidney function in males. Therefore, a mere recommendation of exercise at a clinical examination may lead to the loss of kidney function in males, whereas the weight loss effects of exercise are as yet unclear. Therefore, a recommendation of exercise should be accompanied by instructions on the appropriate type and an amount of exercise provided by healthcare professionals to prevent the loss of kidney function.

There has been no longitudinal study on the relationship between loss of kidney function and short sleep durations in healthy people. In this study, no correlation was observed between the sleep duration and loss of kidney function. According to the results of NHANES, the sleep duration was shorter in the patients with CKD stages 1 or 2 than that in the patients with CKD stages 3 or 4 [8]. However, because these results were obtained in a cross-sectional study, they did not necessarily demonstrate that the risk of loss of kidney function was reduced in people with shorter sleep duration. In a retrospective cohort study of healthy people in Japan, short sleep durations was associated with the incidence of urinary protein excretion [36]. Because sleep disorders include not only short sleep durations but also poor sleep quality and sleep apnea syndrome, which correlate with each other, a longitudinal study is required to evaluate in detail the relationship among incident CKD, the progression of CKD, and sleep disorders.

Our study had several limitations. First, the subjects of this study were healthy, and those with missing values were excluded from the study, which might have resulted in a selection bias. Second, the subjects who were not followed up were excluded from the analysis targets of the longitudinal study. Moreover, the accurate date of onset of loss of kidney function was unclear because data obtained every three years were used. Therefore, the risk of the progression of loss of kidney function might have been underestimated. Third, the types and duration of exercise and the effects of exercise on body weight were not investigated. Therefore, the effects of weight loss on kidney function were not evaluated. Fourth, sleep quality, the use of sleeping pills, and the complication of sleep apnea syndrome were not investigated. Fifth, because this was an observational study, the preventive effect of the improvement in lifestyles on loss of kidney function has remained unclarified. Sixth, because non-alcohol drinkers’ amount of alcohol consumed was treated as 1 g of alcohol/week for ln(alcohol), this approximation may have affected the parameter estimates. Seventh, because we did not investigate what kind of liquor the subjects had, the effects of types of liquor on loss of kidney function could not be evaluated.

## Conclusions

The relationship between lifestyles and loss of kidney function in healthy people was clarified in this study. The small amount of alcohol consumed and high exercise frequency were associated with the increased risk of loss of kidney function in the male groups. These findings suggest that, regardless of obesity, alcohol consumption and exercise may affect loss of kidney function in healthy males.
